# Ionotropic Glutamate Receptors Mediate Inducible Defense in the Water Flea Daphnia pulex

**DOI:** 10.1371/journal.pone.0121324

**Published:** 2015-03-23

**Authors:** Hitoshi Miyakawa, Masanao Sato, John K. Colbourne, Taisen Iguchi

**Affiliations:** 1 National Institute for Basic Biology, 5–1 Higashiyama, Myodaiji, Okazaki, Aichi, Japan; 2 Okazaki Institute for Integrative Bioscience, 5–1 Higashiyama, Myodaiji, Okazaki, Aichi, Japan; 3 Department of Basic Biology, Faculty of Life Science, SOKENDAI (The Graduate University for Advanced Studies), 5–1 Higashiyama, Myodaiji, Okazaki, Aichi, Japan; 4 School of Biosciences, University of Birmingham, Edgbaston, Birmingham, United Kingdom; Federal University of Rio de Janeiro, BRAZIL

## Abstract

Phenotypic plasticity is the ability held in many organisms to produce different phenotypes with a given genome in response to environmental stimuli, such as temperature, nutrition and various biological interactions. It seems likely that environmental signals induce a variety of mechanistic responses that influence ontogenetic processes. Inducible defenses, in which prey animals alter their morphology, behavior and/or other traits to help protect against direct or latent predation threats, are among the most striking examples of phenotypic plasticity. The freshwater microcrustacean *Daphnia pulex* forms tooth-like defensive structures, “neckteeth,” in response to chemical cues or signals, referred to as “kairomones,” in this case released from phantom midge larvae, a predator of *D*. *pulex*. To identify factors involved in the reception and/or transmission of a kairomone, we used microarray analysis to identify genes up-regulated following a short period of exposure to the midge kairomone. In addition to identifying differentially expressed genes of unknown function, we also found significant up-regulation of genes encoding ionotropic glutamate receptors, which are known to be involved in neurotransmission in many animal species. Specific antagonists of these receptors strongly inhibit the formation of neckteeth in *D*. *pulex*, although agonists did not induce neckteeth by themselves, indicating that ionotropic glutamate receptors are necessary but not sufficient for early steps of neckteeth formation in *D*. *pulex*. Moreover, using co-exposure of *D*. *pulex* to antagonists and juvenile hormone (JH), which physiologically mediates neckteeth formation, we found evidence suggesting that the inhibitory effect of antagonists is not due to direct inhibition of JH synthesis/secretion. Our findings not only provide a candidate molecule required for the inducible defense response in *D*. *pulex*, but also will contribute to the understanding of complex mechanisms underlying the recognition of environmental changes, which form the basis of phenotypic plasticity.

## Introduction

Natural environments surrounding living organisms are changing every moment. As a result, phenotypes adaptive under the present circumstances might not continue to be “adaptive” even in the immediate future. Phenotypic plasticity, which is the ability of an organism with a given genome to produce alternative phenotypes in response to environmental stimuli, is an established system found in many animal species that is thought to increase their fitness [1], [2]. In addition to various stimuli, such as temperature, nutrition and population density [1], [3–5], the presence of predators can also trigger morphological and/or life-history changes and, in some cases, induce the so-called “inducible defenses” [6–8]. Understanding the molecular mechanisms underlying how various environmental signals modify ontogenetic processes is regarded as an important subject in the fields of developmental and evolutionary biology.

The freshwater microcrustacean *Daphnia pulex* is a representative species that exhibits an inducible defense. When *D*. *pulex* receives chemical cues (referred to in general as “kairomones”), released in this case from their predator, the phantom midge *Chaoborus* larvae, during embryonic stages, they form tooth-like defensive structures, called “neckteeth,” on the neck region at a post-embryonic instar stage [7]. Exposure of just several hours to the kairomone at the end of embryonic stages is sufficient to induce neckteeth formation, suggesting that the critical period of the kairomone sensing resides mainly just before the transformation to the first instar [9], [10].

Many studies have been performed by our group and others with the goal of elucidating the molecular and developmental underpinnings of the inducible defense of *D*. *pulex*. For example, to date, researchers have found the following: Juvenile hormone pathway mediates neckteeth formation [11], [12]; various morphogenetic factors are expressed in response to the kairomone [11]; and active cell proliferation results in thickened epithelium at the neckteeth region, which is referred to as “crest” [10], [13], [14]. Moreover, genome-wide screening using tiling arrays revealed many differentially expressed genomic regions responding to the kairomone at a post-embryonic instar stage. These factors were thought to be also involved in neckteeth formation [15]. All these developmental phenomena occur following reception of the kairomone signal at the late embryonic stage, in which some neurotransmitter systems are thought to be involved. However, there have been only a couple of studies of the molecular mechanisms underlying this early step of the inducible defense; namely, interesting reports suggesting regulation via the gamma-amino butyric acid (GABA)-ergic neurons and cholinergic neurons [16], [17].

In this study, to help uncover mechanisms underlying sensitive kairomone-reception and rapid developmental-fate determination accomplished during a late embryonic stage, we screened differentially expressed genes within a short time (5 hours) after exposure to the kairomone (before morphological changes begin) using microarray analysis. In addition to identifying the GABA receptors whose regulatory role for neckteeth formation has been suggested [16], we also found that genes coding ionotropic glutamate receptor were significantly up-regulated in response to the kairomone. Furthermore, we demonstrated more reliable evidence that ionotropic glutamate receptors positively regulate neckteeth formation in *D*. *pulex* by exposure experiments using specific agonists and antagonists. These results not only suggest a new factor regulating an early step or steps of the inducible defense in *D*. *pulex*, but also contribute to understanding how animals transmit environmental information into ontogenetic processes.

## Results and Discussion

### Gene screening by microarray

To initiate the study, we used microarray analysis to comprehensively screen for genes differentially expressed in response to the kairomone. The microarray chip used in this study was designed by the *Daphnia* Genomics Consortium and consisted of 134,558 oligonucleotide probes representing 29,569 gene models and 54,416 transcriptionally active regions (hereafter, both will be referred to as “genes”). The kairomone is released only from *Chaoborus* larvae that have been fed daphnids, not from starved *Chaoborus* larvae [14], [18]. To eliminate the effects of differences in water conditions due to the presence or absence of the *Chaoborus*, we used starved-*Chaoborus* water as the control treatment condition. The neckteeth incidences (percentage of neckteeth-forming individuals among all individuals in the experiment) at the first instar under the same treatment as the present study were 7.7% (3/39 juveniles bearing neckteeth) and 82.0% (73/89 juveniles bearing neckteeth) for starved- and fed-*Chaoborus* water, respectively. RNA was extracted at two time-points (1 hr and 5 hr after treatment) and the whole experiment was repeated three times (**S1 Fig**).

Differentially expressed genes affected by the kairomone were analyzed by fitting a linear model to log2-transformed data using R and the Limma package (see Materials and Methods). As a result, 108 and 71 genes were up- and down-regulated, respectively, in response to the kairomone at significant levels (false discovery rate: FDR < 0.05) (**Table 1, S1 Table, S2 Table**). About 80% of these genes (88/108 genes in the up-regulation group, 54/71 genes in the down-regulation group) had not been assigned any gene annotations (**Table 1, S1 Table, S2 Table**). Partially it was because the gene annotation process of *D*. *pulex* is imperfect and still in progress. However, perhaps some of these genes have novel functions in the inducible defense of *D*. *pulex*.

**Table 1:** Up- or down-regulated genes following exposure to the kairomone.

	Gene model + Annotation	Gene model	Unknown transcript	Total
Up-regulation	20	25	63	108
Down-regulation	17	12	42	71

Next, to find functional gene families responding to the kairomone, we performed a gene ontology (GO) enrichment analysis of genes differentially expressed in the presence versus absence of the kairomone. Among the GO terms in the molecular function ontology, a gene cluster having the GO term *ionotropic glutamate receptor activity*, which consist of 65 genes, showed a significant expression change (**Table 2, S3 Table**). Other GO terms which showed low FDR values, i.e., *extracellular ligand-gated ion channel activity*, *excitatory extracellular ligand-gated ion channel activity*, *ligand-gated ion channel activity*, and *gated channel activity*, are effectively equal GO terms to *ionotropic glutamate receptor activity* because these belong to upper-hierarchy GO terms of *ionotropic glutamate receptor activity*. Expression analysis of individual genes also showed the lowest FDR of an ionotropic glutamate receptor among all up-regulated genes and was consistent with the results of the GO enrichment analysis (**S1 Table**). These results strongly suggest that ionotropic glutamate receptors are involved in the inducible defense of *D*. *pulex*. Thus, we next performed exposure experiments using agonists and antagonists of these receptors as described in the following section.

**Table 2:** GO terms in the molecular function ontology significantly enriched among genes with differential responses to the kairomone (FDR < 0.1).

GO ID	GO Term	FDR
GO:0004970	ionotropic glutamate receptor activity	2.95E-10
GO:0005234	extracellular-glutamate-gated ion channel activity	
GO:0008066	glutamate receptor activity	
GO:0005230	extracellular ligand-gated ion channel activity	1.48E-10
GO:0005231	excitatory extracellular ligand-gated ion channel activity	9.83E-11
GO:0015276	ligand-gated ion channel activity	7.38E-11
GO:0022834	ligand-gated channel activity	
GO:0022836	gated channel activity	1.48E-03
GO:0022839	ion gated channel activity	
GO:0004930	G-protein coupled receptor activity	0.0256
GO:0016917	GABA receptor activity	0.0350
GO:0004965	G-protein coupled GABA receptor activity	0.0702
GO:0005261	cation channel activity	0.0877

GABA receptor genes also demonstrated significant up-regulation in both individual gene expression and GO enrichment analyses, although this gene group was associated with a higher FDR as compared with ionotropic glutamate receptor genes (**Table 2, S1 Table**). Neurotransmitter GABA stimulation generally inhibits cellular responses and GABA-ergic antagonists were reported to enhance neckteeth formation of *D*. *pulex* [16]. Although this result was not fully reproduced in a later study [17], GABA-ergic neurons are thought to have an intimate relationship with the inducible defense of *D*. *pulex*.

Among GO terms in the biological process or cellular component, there were no GO terms with a FDR below 0.1.

### Treatment of agonist/antagonist of ionotropic glutamate receptors

Neuronal receptors for glutamate are divided into two groups: metabotropic glutamate receptor, which is a member of the G-protein coupled receptor family, and ionotropic glutamate receptor, which is a member of the ligand-gated ion channel family. The ionotropic glutamate receptors are further divided into three groups whose names are derived from specific agonists, i.e., *N*-methyl-D-aspartic acid (NMDA)-type, (±)-α-amino-3-hydroxy-5-methyl-4-isoxazolepropionic acid (AMPA)-type and kainite-type. These subtypes are expressed mainly in central nervous systems and are involved in various biological processes, including memory and learning, in many animal species [19]. A total of 65 genes had the GO term *ionotropic glutamate receptor activity* (**S3 Table**) but it was largely unclear into what subtype each gene should be grouped (**S2 Fig**). However, as mentioned previously, ionotropic glutamate receptor genes appear to be up-regulated in the presence of the kairomone, suggesting that these receptors positively regulate the inducible defenses of *D*. *pulex*. Therefore, we next investigated the roles of different subtypes of these receptors in inducible defense. We accomplished this using either MK-801, a specific antagonist of NMDA-type glutamate receptors [20], or 2,3-dioxo-6-nitro-1,2,3,4-tetrahydrobenzo[f]quinoxaline-7-sulfonamide (NBQX), a specific antagonist of non-NMDA-type (AMPA-type and kainate-type) glutamate receptors [21], during embryonic and postembryonic development of daphnids.

**Fig 1:**
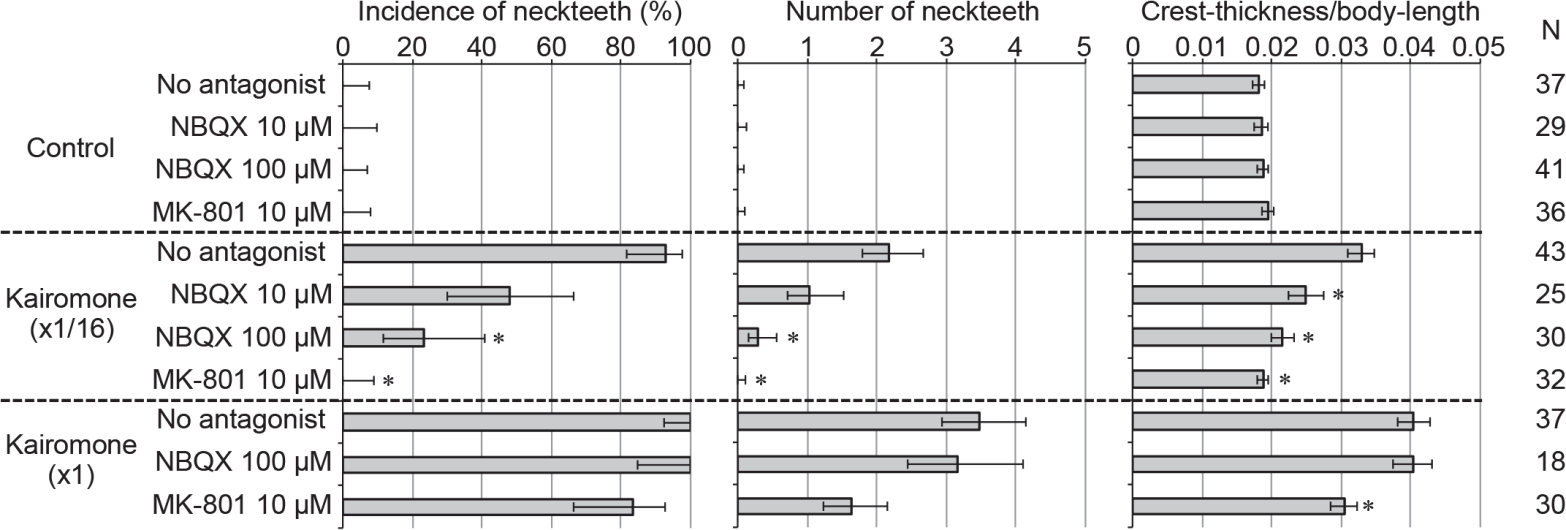
Effect of ionotropic glutamate receptor antagonists on the three different indicators of the defensive morph. The indicators are incidence of neckteeth (left), average number of neckteeth (center), and crest thickness normalized to body-length (right). Error bars indicate 95% confidence intervals. Asterisks indicate significant differences (p < 0.01) as compared with *Daphnia* exposed to the same kairomone conditions without antagonists (-). N: number of specimens.

Exogenous treatment with either antagonist (*i*.*e*. MK-801 or NBQX) demonstrated a clear inhibitory effect on kairomone-dependent neckteeth formation (**Fig. 1**). The *D*. *pulex* clone used in this study shows very strong induction of neckteeth even in response to low levels of the kairomone (kairomone (x1/16)). In the presence of both kairomone (x1/16) and MK-801, none of daphnids formed neckteeth. Crest-thickness is a more sensitive indicator of the defensive morph, as the crest becomes thicker even if the kairomone concentration does not reach levels sufficient to induce neckteeth spines. In the presence of kairomone (x1/16) and MK-801, crest thickness was remained at control levels. NBQX also showed similar inhibitory effect on the inducible defense, although its activity was somewhat lower than that of MK-801. In addition, exposure to a higher concentration of the kairomone (kairomone (x1)) compensated for inhibition of neckteeth formation by the chemical antagonists (**Fig. 1**). These results strongly suggest that ionotropic glutamate receptor activity is necessary for neckteeth formation of *D*. *pulex*, consistent with the hypothesis derived from differential gene expression induced by kairomone. Moreover, neckteeth formation might be mediated mainly by NMDA-type receptors, as evidence by the stronger effect of MK-801 than NBQX, although some contributions of non-NMDA-type receptors should be considered.

**Fig 2:**
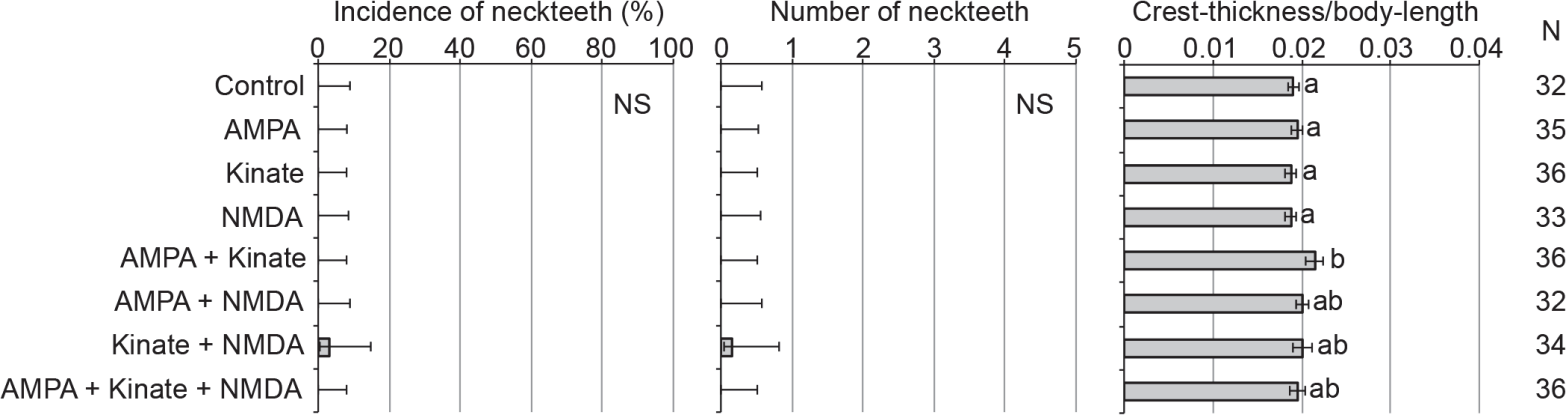
Effect of ionotropic glutamate receptor agonists on the three different indicators of the defensive morph. The indicators are incidence of neckteeth (left), average number of neckteeth (center), and crest thickness normalized to body-length (right). The final concentration of all chemicals used in this experiment was 10 μM. Error bars indicate 95% confidence intervals. Different letters indicate significant differences (p < 0.01). N: number of specimens.

On the other hand, even when we applied specific agonists of each receptor subtype (i.e., NMDA, AMPA and kainate), defensive traits were not induced except for a slight increase in crest thickness following co-exposure to multiple agonists (**Fig. 2**). The activation of ionotropic glutamate receptors is not expected to be sufficient for neckteeth formation itself. Another possibility is that the ionotropic glutamate receptor(s) responsible for neckteeth formation is not categorized into any of the three subtypes and is not susceptible to these agonists. In fact, molecular phylogenetic analysis revealed that most of these receptor genes found in the *D*. *pulex* genome form a clade that is independent from known NMDA and non-NMDA receptors (**S2 Fig**).

None of the chemicals induced any toxicity, such as would be indicated by a decrease in the survival rate or abnormal development, under the concentrations used in these experiments, although treatment with a higher concentration (100 μM) of MK-801 severely reduced the survival rate (data not shown).

### Hierarchical relationship between ionotropic glutamate receptor and JH signaling

**Fig 3:**
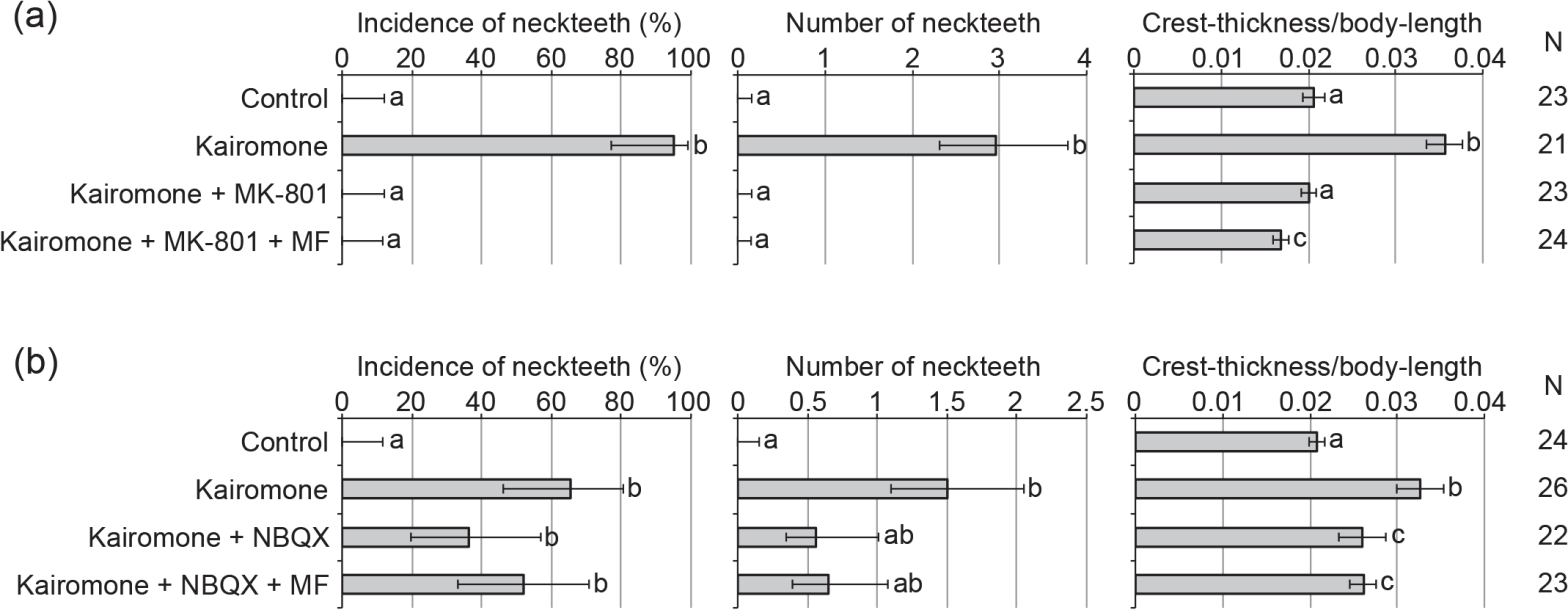
Effect of coexposure of methyl farnesoate (MF) and MK-801 (a) or NBQX (b) on the three different indicators of defensive morph. The indicators are incidence of neckteeth (left), average number of neckteeth (center), and crest thickness normalized to body-length (right). Kairomone (x1/16) was used for neckteeth induction. The final concentrations of chemicals used in this experiment were as follows: 10 μM of MK-801; 100 μM of NBQX; 100 μg/L (400 nM) of MF. Error bars indicate 95% confidence intervals. Different letters indicate significant differences (p < 0.01). N: number of specimens.

Juvenile hormone (JH) is a key endocrine factor playing various physiological roles in arthropods, including insects and crustaceans [22], [23]. We previously reported that JH positively regulates neckteeth formation of *D*. *pulex* downstream of kairomone reception, but that activation of JH signaling alone cannot induce neckteeth formation [12]. In insects, NMDA-type ionotropic glutamate receptors expressed in the corpora allata, where JH is synthesized, are known to positively regulate JH secretion from the corpora allata [24–27]. Although a JH-synthesizing organ(s) have not yet been identified in daphnids, there is a possibility that inhibition of neckteeth formation by ionotropic glutamate receptor antagonists shown in the present study is a result of the inhibition of synthesis and/or secretion of JH. To test this possibility, we first asked if inhibition of neckteeth formation by antagonists is rescued by additional exposure to a crustacean JH, methyl farnesoate (MF). MF did not recover any defensive traits (i.e., no change in incidence, number of neckteeth, or crest-thickness) when introduced along with either MK-801 or NBQX treatments (**Fig. 3**). This result suggests that the ionotropic glutamate receptor antagonists inhibit neckteeth formation not via simple inhibition of JH synthesis and/or secretion, but perhaps due to effects on earlier steps. As for other examples of phenotypic plasticity, in the process of neckteeth formation in *D*. *pulex*, individuals receiving the kairomone signal are expected to undergo physiological alterations mediated by endocrine systems such as JH signaling, followed by expression of many morphogenetic factors leading to actual morphological changes [11]. Because ionotropic glutamate receptors are expressed in the central nervous systems and are involved in neurotransmission [19], these receptors might mediate the initial step of the inducible defense, i.e., kairomone reception, such as by cooperating with GABA-ergic and cholinergic systems previously suggested to regulate neckteeth formation [16], [17]. However, the present study does not reject the possibility of regulation of JH synthesis/secretion by ionotropic glutamate receptors in *D*. *pulex*. A more general characterization of ionotropic glutamate receptors in crustaceans will contribute to our understanding of the complex roles of receptors in this receptor family [28].

We demonstrated that the transcriptome approach is a powerful and useful method to shed light on the molecular mechanisms underlying the inducible defense of *D*. *pulex*. Although we identified ionotropic glutamate receptors in the study, they do not appear to be sufficient for neckteeth formation. Uncharacterized genes identified in the microarray analysis may include other essential factors (**Table 1, S1 Table, S2 Table**). We recently established that RNAi can be performed in *Daphnia* [29], [30], providing a possible approach for functional analysis of these candidate genes. Another possible approach is isolation and determination of the molecular make-up of the *Chaoborus* kairomone, which remains unidentified. If it was identified, we might more easily address the most unclear steps of the inducible defense, i.e., kairomone reception and signal transduction. More advanced genomic information is available for *D*. *pulex* as compared with other crustaceans [15]. As we learn more about inducible defense in this system, then, *D*. *pulex* might emerge as a model system uniquely suited to the study of phenotypic plasticity with relevance to the fields of ecology and evolution.

## Materials and Methods

### Daphnids

The clone of *D*. *pulex* used in the experiments was collected from a pool at Maeda Forest Park in Sapporo in 2009. *D*. *pulex* is commonly living in Japan and is not an endangered or protected species. Maeda Forest Park is not a protected area but a common park in the urban area of Sapporo city. Therefore, no specific permissions were required. The clone was reared in the laboratory at 20°C in aged tap water and fed unicellular green algae (Chlorella Industry Co. Ltd, Fukuoka, Japan) over generations in a temperature- and photocycle-controlled incubator (20°C, 16-h light/8-h dark).

### Kairomone medium

Fourth-instar *Chaoborus flavicans* larvae were collected from a pond at the National Institute for Environmental Studies (NIES), Tsukuba, Ibaraki, Japan. *Chaoborus* is not an endangered or protected species. Sample collection was performed with permission of NIES. *Chaoborus* larvae were reared in dechlorinated tap water at a density of 10–15 larvae/L for more than 7 days in a temperature- and photoperiod-controlled incubator and fed *D*. *pulex* daily with sufficient. After 7 days, the water was filtered using Whatman GF/C filters (Whatman, London, UK) to remove any daphnid juveniles and particulate matter (> 1 μm) before being stored in plastic bottles at -20°C. At the time of the experiments, the water samples were thawed at 20°C and used as a rearing medium for *D*. *pulex* (fed *Chaoborus*-conditioned medium). In this study, the undiluted kairomone water was referred to as the “x1” kairomone concentration (indicated as “kairomone (x1)”). We then prepared a 1/16 dilution of the kairomone (x1) water using filtered tap water (indicated as “kairomone (x1/16)”). Starved *Chaoborus*-conditioned medium was prepared by the same way as fed *Chaoborus*-conditioned medium except for feeding with *D*. *pulex*.

### RNA extraction

Six hundred two pink-eye stage embryos, i.e., the late stage of the kairomone-sensitive period [9], [10], were removed from maternal brood chambers and treated with either fed or starved *Chaoborus*-conditioned media (**S1 Fig**). After incubation at 20°C for 1 hr, about half the animals in each treatment culture (150 embryos each) were collected and total RNA was extracted using an RNAqueous-Micro Kit (Life Technologies, Gaithersburg, MD, USA). After 4 hr additional incubation, the remaining animals were collected and total RNA was extracted (**S1 Fig**). This procedure was repeated three times independently, resulting in 12 samples (4 conditions x 3 replicates) that were used for microarray analysis. Additional embryos were used to confirm the effectiveness of kairomone treatment by checking the incidence of neckteeth in first-instar juveniles.

### Microarray

Microarray analysis using NimbleGen array (Roche NimbleGen, Madison, WI, USA) was performed according to the manufacturer’s instructions and the technical note “Roche cDNA Synthesis System for use with NimbleGen Gene Expression Microarray”. One micro-gram total RNA of each sample was used for analysis. The slide was scanned by a microarray scanner G2565CA (Agilent Technologies, Santa Clara, CA, USA). To increase the accuracy of signal measurements by the scanner, we used two different photo-multiplier tube laser power settings (10% and 100%) for the target signal. Raw data were extracted as pair files using NimbleScan software version 2.6 (Roche NimbleGen) and combined to obtain a raw expression value for each probe [31], [32]. The platform can be found on the Gene Expression Omnibus, accession number GSE63275.

To identify genes that are up- or down-regulated in response to predatory kairomone, log_2_-transformed expression values were fitted to a linear model for each probe using the Limma package [33]:

$$E_{ijk}\sim K_{i}+T_{j}+R_{k}+\varepsilon_{ijk}$$

where *E*, *K*, *T*, *R*, and *ε* are log_2_-transformed expression value, kairomone treatment, developmental time, replicates, and residuals, respectively. The false discovery rate (FDR) was calculated using Storey’s method [34]. Gene models, annotations and gene ontology (GO) terms were assigned to each probe according to the genome project data of *D*. *pulex* [15]. GO enrichment analysis was performed using ErmineJ overrepresentation analysis [35]. Among 21,987 genes bearing at least one GO term, GO subsets containing between 5 and 150 genes were included in the analysis. GO subsets with a Benjamini-Hochberg FDR < 0.1 were considered significant [36].

### Induction of defensive morph and chemical treatment

The defensive phenotype with neckteeth was induced as described previously with slight modifications [9], [12]. Early stage embryos (egg chorion present) were removed from brood chambers of fully-grown adults having 20–30 eggs, and placed in a Petri dish. Individual embryos were then randomly assigned to separate wells of a 48-well plate. Each well contained 500 μl of either kairomone (x1), kairomone (x1/16) or filtered tap water as control, which contained experimental chemical(s) or solvent alone. After incubation for 72 h at 20°C, second-instar juveniles were collected and observed under a microscope (CKX41, Olympus, Tokyo, Japan) for analysis of phenotypes. Juvenile instar was determined precisely by checking for a cast-off exoskeleton.

In this study, we used three agonists of ionotropic glutamate receptors, *N*-methyl-D-aspartic acid (NMDA) (≥98%; Sigma-Aldrich, St. Louis, MO, USA), (±)-α-amino-3-hydroxy-5-methyl-4-isoxazolepropionic acid (AMPA) (≥98%; Sigma-Aldrich) and kainic acid *n*-hydrate (≥98%; Wako, Osaka, Japan); two antagonists, (+)-MK-801 hydrogen maleate (≥98%; Sigma-Aldrich) and 2,3-dioxo-6-nitro-1,2,3,4-tetrahydrobenzo[f]quinoxaline-7-sulfonamide (NBQX) disodium salt hydrate (≥98%; Sigma-Aldrich); and a crustacean juvenoid, methyl farnesoate (MF) (≥95%; Echelon Bioscience, Salt Lake City, UT, USA). Each agonist and antagonist of ionotropic glutamate receptors was dissolved in water and stored as a 10 mM stock solution. Stock solutions were then diluted at least 100-fold into kairomone or control media at the time of exposure. MF was dissolved in ethanol and stored as a 10 mg/mL stock solution, and diluted at least 100,000-fold into kairomone or control media at the time of exposure. The final concentration of MF was 100 μg/L (400 nM).

### Analysis of phenotypes

Based on our previous study [12], morphological differences depending on treatment were evaluated by looking for the following three traits: 1) incidence of neckteeth, 2) number of neckteeth, 3) crest-thickness normalized to body length. Comparisons of the incidence and average number of neckteeth were performed using the Fisher’s exact test with Holm correction, and the Kruskal-Wallis test followed by Scheffe’s post-hoc test, respectively. Comparison of normalized crest-thickness was performed by ANOVA followed by the post hoc Tukey-Kramer test. All statistical analyses were performed using R 2.15.2 [37] and/or Excel 2011 (Microsoft Corp., Redmond, WA, USA) with the add-in software Statcel 2 [38].

## Supporting Information

S1 Fig Schematic view of how *Daphnia* RNA was prepared for microarray analysis. (DOCX)

S1 Table Genes up- or down-regulated in response to predatory kairomone as determined by microarray analysis (FDR < 0.05). (XLSX)

S2 Table Uncharacterized transcripts up- or down-regulated in response to predatory kairomone as determined by microarray analysis (FDR < 0.05). (XLSX)

S3 Table Gene models containing the GO term *ionotropic glutamate receptor activity* (GO:0004970). (XLSX)

S2 Fig Molecular phylogenetic reconstruction of 65 ionotropic glutamate receptors of *Daphnia pulex* and known homologs of model organisms, Rattus norvegicus and *Drosophila melanogaster*. *D*. *pulex* gene models are denoted by Protein IDs in wFleaBase (http://wfleabase.org/). The compressed subtree (black triangle) contains 52 monophyletic gene models of *D*. *pulex*. The amino acid sequences were aligned using the MEGA6 software MUSCLE method with the default options. A maximum likelihood tree was constructed from these alignments using a JTT model with bootstrap analyses of 500 replicates along with complete deletion options (60 amino acid positions). Branches with *bootstrap* support > 50% *are indicated by* numbers at nodes. *The scale bar represents the number of substitutions per site*. *D*. *pulex gene models used in this analysis are listed in* **S3 Table**. *Accession numbers of rat and fly sequences are as follows*. *Rattus norvegicus NMDA receptor*: *NR1 (CAA44914*.*1)*, *NR2A (AAC03565*.*1)*, *NR2B (AAA41714*.*1)*, *NR2C (AAA41713*.*1)*, *NR2D (BAA02500*.*1)*, *NR3A (AAA99501*.*1)*. *R*. *norvegicus AMPA receptor*: *GluR1 (AAA41243*.*2)*, *GluR2 (AAA41244*.*1)*, *GluR3 (AAA41245*.*1)*, *GluR4 (AAA41246*.*1)*. *R*. *norvegicus kainate receptor*: *GluR5 (P22756*.*3)*, *GluR6 (P42260*.*2)*, *GluR7 (P42264*.*1)*, *KA1 (1712321A)*, *KA2 (AAA17831*.*1)*. *R*. *norvegicus glutamate receptor delta*: *GRID1 (CAA78936*.*1)*, *GRID2 (CAA78937*.*1)*. *Drosophila melanogaster NMDA receptor*: *NR1 (AAF52016*.*1)*, *NR2 (AAF45640*.*2)*. *D*. *melanogaster AMPA receptor*: *GluR1 (AAA28575*.*1)*.
(DOCX)
