# Supplementary figures and images for: Ionotropic Glutamate Receptors Mediate Inducible Defense in the Water Flea Daphnia pulex
Source: PLoS One. 2015 Mar 23;10(3):e0121324. doi: 10.1371/journal.pone.0121324 (PMC4370714; doi:10.1371/journal.pone.0121324)

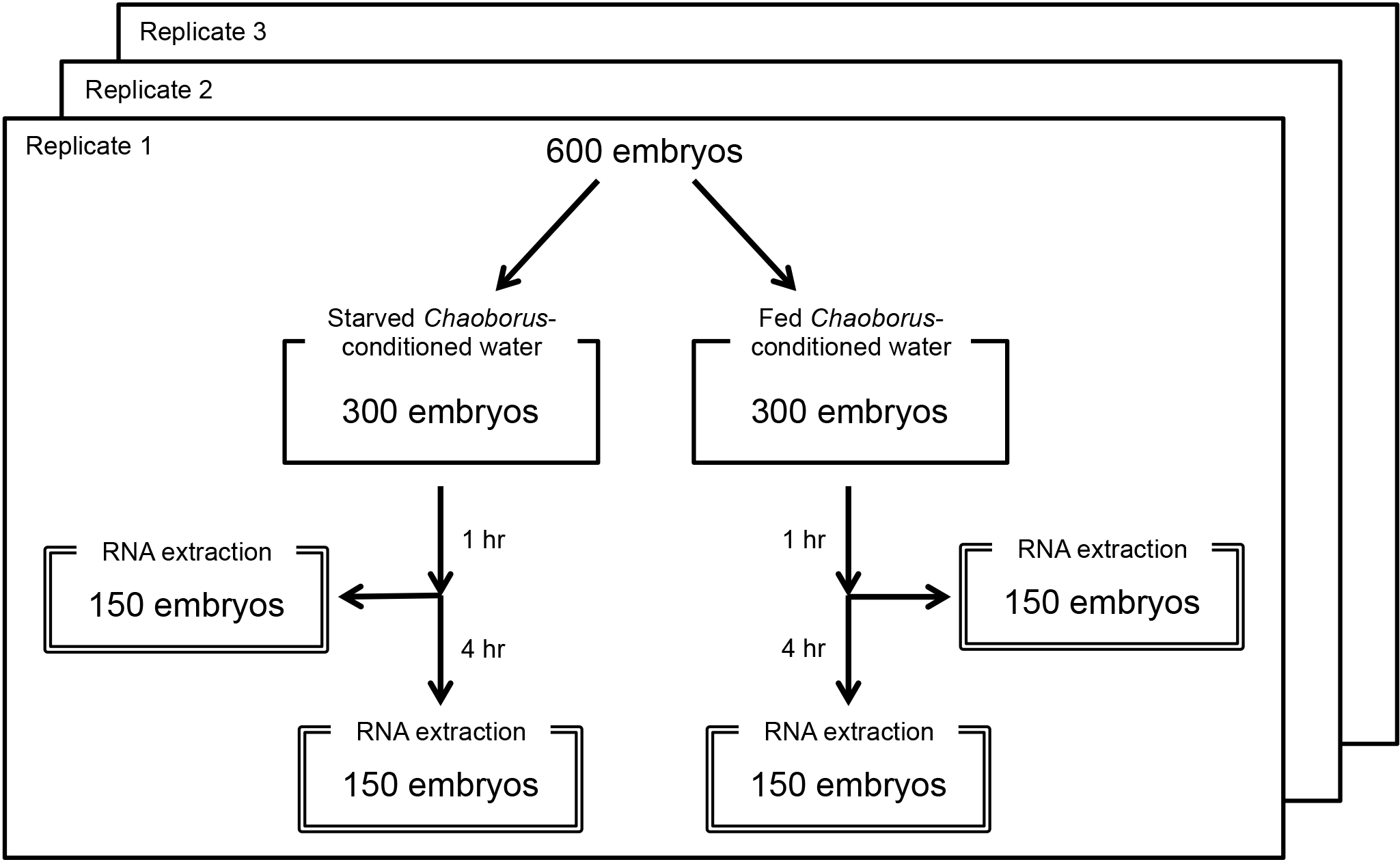


**S1_Fig.** Schematic view of how *Daphnia* RNA was prepared for microarray analysis.

Supplement: S1 Fig — (DOCX) [file pone.0121324.s001.docx]
