# Supplementary material for: Ionotropic Glutamate Receptors Mediate Inducible Defense in the Water Flea Daphnia pulex
Source: PLoS One. 2015 Mar 23;10(3):e0121324. doi: 10.1371/journal.pone.0121324 (PMC4370714; doi:10.1371/journal.pone.0121324)
Supplement: S2 Fig — D. pulex gene models are denoted by Protein IDs in wFleaBase (http://wfleabase.org/). The compressed subtree (black triangle) contains 52 monophyletic gene models of D. pulex. The amino acid sequences were aligned using the MEGA6 software MUSCLE method with the default options. A maximum likelihood tree was constructed from these alignments using a JTT model with bootstrap analyses of 500 replicates along with complete deletion options (60 amino acid positions). Branches with bootstrap support > 50% are indicated by numbers at nodes. The scale bar represents the number of substitutions per site. D. pulex gene models used in this analysis are listed in S3 Table. Accession numbers of rat and fly sequences are as follows. Rattus norvegicus NMDA receptor: NR1 (CAA44914.1), NR2A (AAC03565.1), NR2B (AAA41714.1), NR2C (AAA41713.1), NR2D (BAA02500.1), NR3A (AAA99501.1). R. norvegicus AMPA receptor: GluR1 (AAA41243.2), GluR2 (AAA41244.1), GluR3 (AAA41245.1), GluR4 (AAA41246.1). R. norvegicus kainate receptor: GluR5 (P22756.3), GluR6 (P42260.2), GluR7 (P42264.1), KA1 (1712321A), KA2 (AAA17831.1). R. norvegicus glutamate receptor delta: GRID1 (CAA78936.1), GRID2 (CAA78937.1). Drosophila melanogaster NMDA receptor: NR1 (AAF52016.1), NR2 (AAF45640.2). D. melanogaster AMPA receptor: GluR1 (AAA28575.1). (DOCX) [file pone.0121324.s005.docx]

**S5_Fig.** Molecular phylogenetic reconstruction of 65 ionotropic glutamate receptors of *Daphnia pulex* and known homologs of model organisms, Rattus norvegicus and *Drosophila melanogaster*. *D. pulex* gene models are denoted by Protein IDs in wFleaBase (http://wfleabase.org/). The compressed subtree (black triangle) contains 52 monophyletic gene models of *D. pulex*. The amino acid sequences were aligned using the MEGA6 software MUSCLE method with the default options. A maximum likelihood tree was constructed from these alignments using a JTT model with bootstrap analyses of 500 replicates along with complete deletion options (60 amino acid positions). Branches with bootstrap support > 50% are indicated by numbers at nodes. The scale bar represents the number of substitutions per site. D. pulex gene models used in this analysis are listed in **S4_Table**. Accession numbers of rat and fly sequences are as follows. Rattus norvegicus NMDA receptor: NR1 (CAA44914.1), NR2A (AAC03565.1), NR2B (AAA41714.1), NR2C (AAA41713.1), NR2D (BAA02500.1), NR3A (AAA99501.1). R. norvegicus AMPA receptor: GluR1 (AAA41243.2), GluR2 (AAA41244.1), GluR3 (AAA41245.1), GluR4 (AAA41246.1). R. norvegicus kainate receptor: GluR5 (P22756.3), GluR6 (P42260.2), GluR7 (P42264.1), KA1 (1712321A), KA2 (AAA17831.1). R. norvegicus glutamate receptor delta: GRID1 (CAA78936.1), GRID2 (CAA78937.1). Drosophila melanogaster NMDA receptor: NR1 (AAF52016.1), NR2 (AAF45640.2). D. melanogaster AMPA receptor: GluR1 (AAA28575.1).
